# Mutually Exclusive Roles of SHARPIN in Integrin Inactivation and NF-κB Signaling

**DOI:** 10.1371/journal.pone.0143423

**Published:** 2015-11-23

**Authors:** Nicola De Franceschi, Emilia Peuhu, Maddy Parsons, Sami Rissanen, Ilpo Vattulainen, Marko Salmi, Johanna Ivaska, Jeroen Pouwels

**Affiliations:** 1 Turku Centre for Biotechnology, University of Turku, Turku, Finland; 2 Medical Biotechnology, VTT Technical Research Centre of Finland, Turku, Finland; 3 Randall Division of Cell and Molecular Biophysics, King's College London Guy's Campus, London, United Kingdom; 4 Department of Physics, Tampere University of Technology, Tampere, Finland; 5 MEMPHYS-Center for Biomembrane Physics, University of Southern Denmark, Odense, Denmark; 6 MediCity Research Laboratory, University of Turku, Turku, Finland; 7 Department of Medical Microbiology and Immunology, University of Turku, Turku, Finland; 8 Department of Biochemistry and Food Chemistry, University of Turku, Turku, Finland; University of Bergen, NORWAY

## Abstract

SHANK-associated RH domain interactor (SHARPIN) inhibits integrins through interaction with the integrin α-subunit. In addition, SHARPIN enhances nuclear factor-kappaB (NF-κB) activity as a component of the linear ubiquitin chain assembly complex (LUBAC). However, it is currently unclear how regulation of these seemingly different roles is coordinated. Here, we show that SHARPIN binds integrin and LUBAC in a mutually exclusive manner. We map the integrin binding site on SHARPIN to the ubiquitin-like (UBL) domain, the same domain implicated in SHARPIN interaction with LUBAC component RNF31 (ring finger protein 31), and identify two SHARPIN residues (V267, L276) required for both integrin and RNF31 regulation. Accordingly, the integrin α-tail is capable of competing with RNF31 for SHARPIN binding in vitro. Importantly, the full SHARPIN RNF31-binding site contains residues (F263A/I272A) that are dispensable for SHARPIN-integrin interaction. Importantly, disrupting SHARPIN interaction with integrin or RNF31 abolishes SHARPIN-mediated regulation of integrin or NF-κB activity, respectively. Altogether these data suggest that the roles of SHARPIN in inhibiting integrin activity and supporting linear ubiquitination are (molecularly) distinct.

## Introduction

Integrins are heterodimeric transmembrane receptors composed of an α- and a β-subunit that mediate cell interaction with the extracellular matrix and neighboring cells. Binding of extracellular ligands or intracellular integrin activators, such as TALINs or FERMTs (also known as kindlins), induces a pronounced conformational change in the integrin heterodimer. This conformational switch results in integrin activation and the formation of a large macromolecular complex at integrin cytoplasmic tails that connects the adhesion site to the actin cytoskeleton and activates a plethora of intracellular signaling pathways [1]. Deregulated integrin activity has been implicated in many human pathologies, including immune diseases, skin blistering, bleeding disorders and cancer (reviewed in [1–3]). We previously identified SHANK-associated RH domain interactor (SHARPIN) as an important integrin inactivator, which interacts directly with the conserved region of the α-integrin cytoplasmic domain and prevents recruitment of TALIN and FERMTs to the integrin beta 1 (ITGB1) cytoplasmic tail [4]. More recently, we showed that SHARPIN is an important regulator of lymphocyte migration as it inactivates integrin alpha L-integrin beta 2 (ITGAL-ITGB2, also known as LFA-1) to support uropod detachment in polarized lymphocytes [5].

Nuclear factor-kappaB (NF-κB) is an oncogenic and pro-inflammatory transcription factor that mediates the cellular response to a wide array of stimuli. SHARPIN was identified as an essential component of the Linear Ubiquitination Assembly Complex (LUBAC) [6–8], which stimulates canonical NF-κB signaling in response to cytokines, bacteria and genotoxic stress [9].

These studies demonstrate that SHARPIN regulates at least two important, yet seemingly distinct cellular pathways: integrin activation [4] and canonical NF-κB signaling [6–8]. Nevertheless, the relationship between these two SHARPIN functions has remained obscure. To investigate this further we performed detailed structure-function studies on the contribution of SHARPIN to integrin and LUBAC regulation. We find that integrins, similar to the catalytic subunit of LUBAC (RNF31; ring finger protein 31, also known as HOIL-1-Interacting Protein (HOIP)) [6], bind the conserved central ubiquitin-like domain (UBL) of SHARPIN. Furthermore, mutational analyses, based on a 3D model of the SHARPIN UBL domain, revealed that integrin and RNF31 interact with partially overlapping regions within the UBL domain of SHARPIN. Thus, RNF31 and integrin bind to SHARPIN in a mutually exclusive manner, suggesting that the roles of SHARPIN in inhibiting integrin activity and supporting linear ubiquitination are molecularly distinct.

## Results

### The ubiquitin-like domain (UBL) of SHARPIN is the binding site for integrin α-chains

Direct interaction between SHARPIN and integrin α-tails or RNF31 is essential for SHARPIN-mediated integrin inhibition [4] and LUBAC function [6–8], respectively. RNF31 binds the conserved central UBL domain of SHARPIN [6], but the integrin binding region of SHARPIN has not been identified. As the SHARPIN-binding region is conserved within all α-integrins and as SHARPIN inhibits both ITGB1s and leukocyte-specific ITGB2s [4,5], we designed experiments to analyze SHARPIN-integrin interaction using different integrin heterodimers and cell types to ensure generality of our findings.

**Fig 1:**
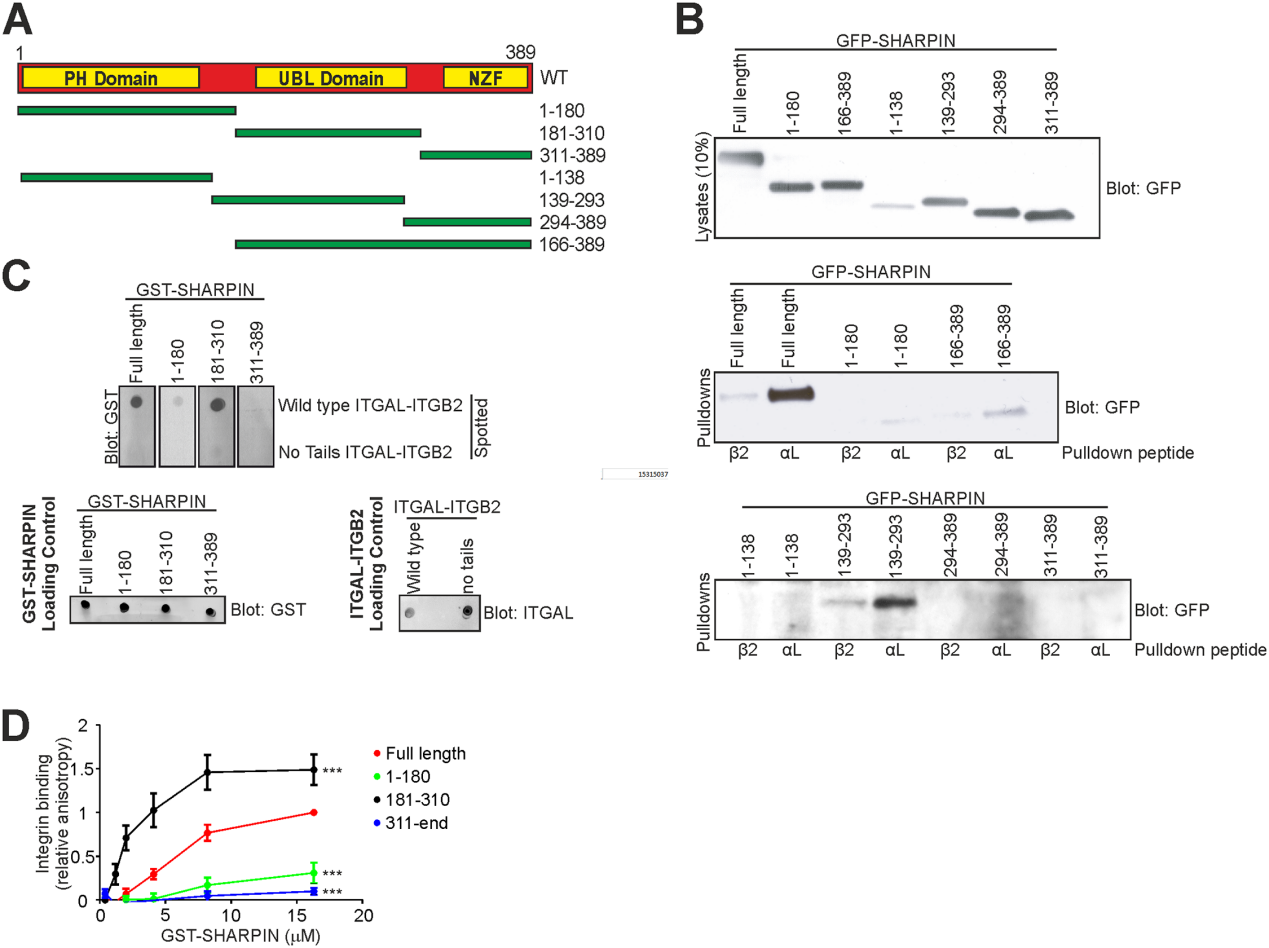
The UBL domain of SHARPIN mediates binding to integrin. (A) Schematic representation of SHARPIN with its functional domains and the SHARPIN fragments used in this study. (B) Pull-down experiments to determine the interaction between GFP-SHARPIN (full-length or fragments) and peptides corresponding to the cytoplasmic domain of ITGAL and ITGB2. (C) Far-Western analysis of GST-SHARPIN (full-length or fragments) binding to full-length ITGAL-ITGB2 or ITGAL-ITGB2 lacking both cytoplasmic tails. Loading controls for GST-SHARPIN (full-length or fragments) and both ITGAL-ITGB2s are shown. (D) Fluorescence polarization-based titration of GST-SHARPIN (full-length or fragments) binding to an integrin peptide corresponding to the conserved domain within the cytoplasmic tail of ITGA2. Average normalized binding curves are shown (mean ± s.e.m. ***: p<0.001).

SHARPIN has three conserved functional domains; the N-terminal pleckstrin homology (PH) superfold that mediates homomultimerization [10,11], the central UBL domain and the C-terminal NPL4 zinc finger domain (NZF) that is required for LUBAC function [6]. To map the integrin binding site on SHARPIN, fragments of the SHARPIN coding sequence, containing one or more functional domains flanked by additional regions (Fig 1A), were cloned into GFP and GST expression vectors. A pull-down assay was performed to assess binding of the indicated GFP-SHARPIN fragments to biotinylated peptides corresponding to the cytoplasmic domain of ITGAL [5], using an ITGB2 peptide as a negative control. GFP-SHARPIN^181-310^ was not included due to very low expression levels. Interestingly, the ITGAL tail was found to interact with all SHARPIN fragments containing the central UBL domain, but not the N- or C-terminal fragments, suggesting that the UBL domain contains the integrin-binding site (Fig 1B). Far-Western analyses using GST-SHARPIN^WT^ or GST-SHARPIN fragments, and recombinant full-length ITGAL-ITGB2 (Fig 1C; ITGAL-ITGB2 lacking cytoplasmic tails was used as a negative control), in addition to fluorescence polarization titrations between GST-SHARPIN (WT or fragments) and an ITGA2 peptide (Fig 1D) confirmed that SHARPIN binds integrin α-tails via its UBL domain. Thus, integrins and RNF31 both interact with the UBL domain of SHARPIN.

### Fine-mapping of the integrin binding site on SHARPIN

**Fig 2:**
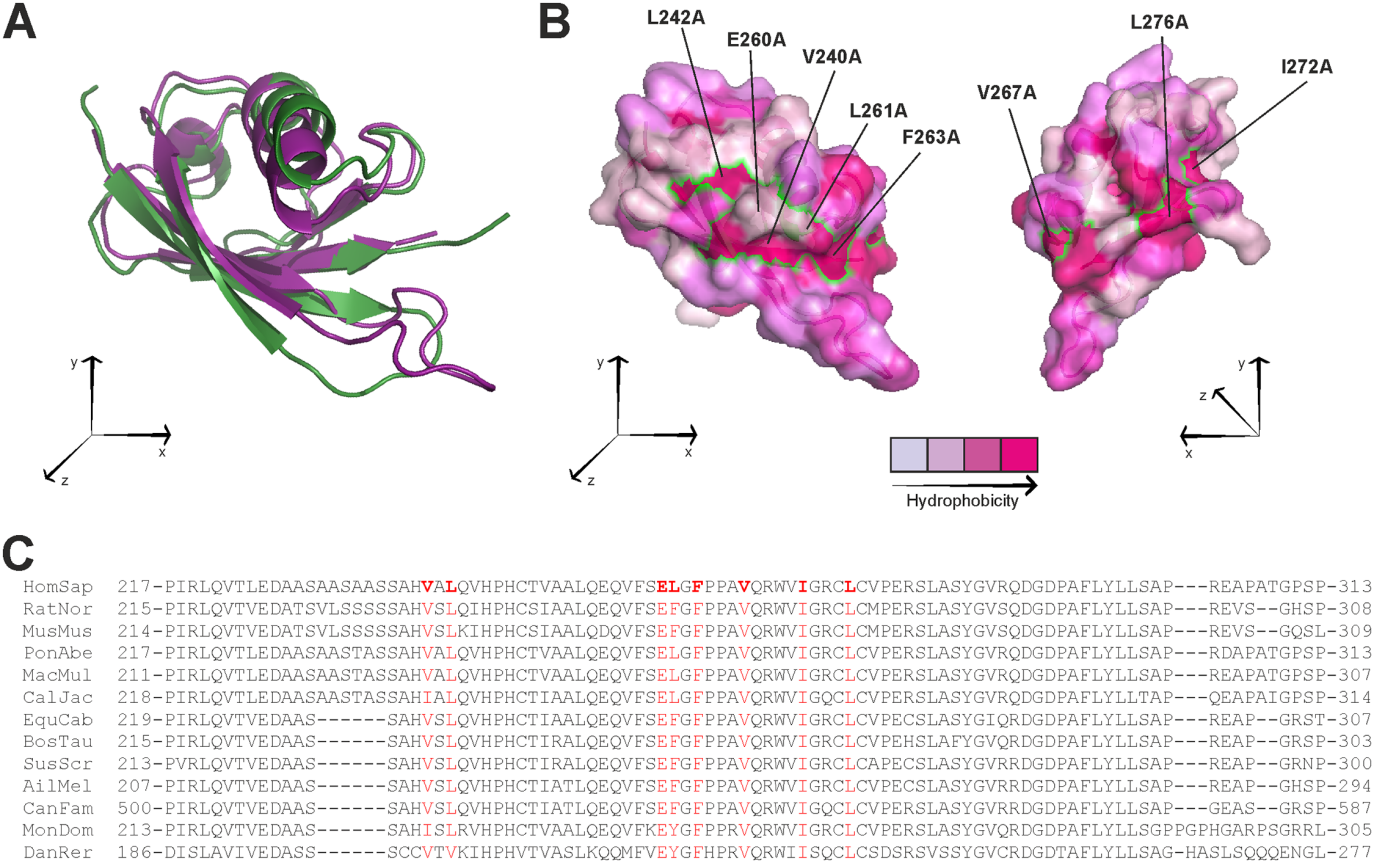
Designing SHARPIN mutants using a SHARPIN UBL domain model. (A) Superposition of the SHARPIN UBL domain model backbone (purple) with the UBL domain of HOIL1L (Green, PDB accession code: 4DBG). (B) Surface representation of the SHARPIN UBL model. The surface is color-coded according to residue hydrophobicity from light to deep purple. Footprints of residues mutated in this study are indicated as green outlines. (C) Alignment of part of the SHARPIN UBL domain across different species. Conserved residues mutated in this study are indicated in red.

As structural data on the SHARPIN UBL domain are currently unavailable, the Swiss Model Server [12] was used to create a homology model in order to map the putative integrin binding site within the UBL domain. Superposition of the SHARPIN UBL domain model backbone with the UBL domain of HOIL1L (longer isoform of RanBP-Type And C3HC4-Type Zinc Finger-Containing 1 (RBCK1), the third member of LUBAC in addition to SHARPIN and RNF31) (Fig 2A) and subsequent surface hydrophobicity analysis (Fig 2B) to identify putative binding sites [13], revealed specific evolutionary conserved hydrophobic surface residues within SHARPIN (Fig 2B and 2C). These residues were chosen for alanine scanning mutational analysis, resulting in three double and three single SHARPIN mutants; SHARPIN^V240A/L242A^, SHARPIN^E260A/L261A^, SHARPIN^L261A/F263A^, SHARPIN^V267A^, SHARPIN^I272A^ and SHARPIN^L276A^. SHARPIN^I272A^ was shown previously to abolish RNF31 binding [6].

**Fig 3:**
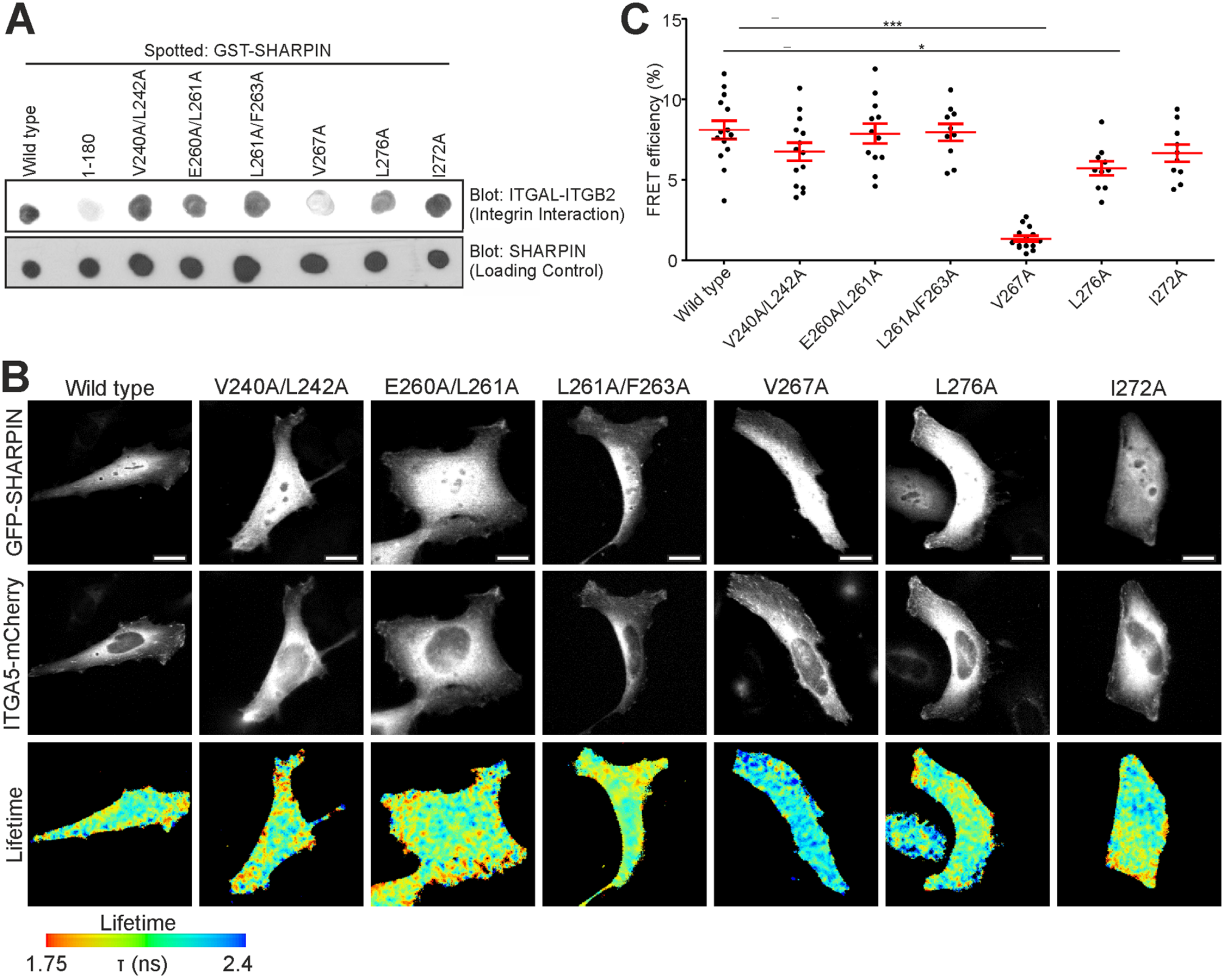
Fine mapping of the integrin binding site in SHARPIN. (A) Interaction of different GST-SHARPIN (WT and all point mutants) with full-length ITGAL-ITGB2 was determined using Far-Western assays. GST-SHARPIN^1-180^ was used as negative control. (B) HEK293 cancer cells, overexpressing WT or mutant GFP-SHARPIN in combination with ITGA5–mCherry, subjected to FRET analysis by FLIM. Fluorescence lifetimes, mapping spatial FRET in cells, are depicted using a pseudo-color scale (blue, normal lifetime; red, FRET (reduced lifetime)). Scale bars: 10 μm. (C) Quantification of FRET efficiency for all mutants (n ≥ 12 cells). All numerical data are mean ± s.e.m. ***: p<0.001, *: p<0,05.

Far-Western analysis revealed clearly reduced ITGAL-ITGB2 binding by SHARPIN^V267A^ compared to SHARPIN^WT^ (Fig 3A), while ITGAL-ITGB2 binding by SHARPIN^L276A^ was only mildly affected. These observations were confirmed using fluorescence lifetime imaging microscopy (FLIM) measurements of fluorescence resonance energy transfer (FRET) between GFP-SHARPIN (WT or mutants) and ITGA5-mCherry [4]. Compared to GFP-SHARPIN^WT^, GFP-SHARPIN^V267A^ showed strongly reduced FRET, whereas SHARPIN^L276A^ exhibited a small but significant reduction in FRET efficiency, as evidenced by the representative FRET lifetime images and cumulative FRET efficiency data (Fig 3B and 3C). None of the other mutations, including SHARPIN^I272A^, affected FRET efficiency (Fig 3B and 3C), indicating that these residues were not required for integrin binding. Importantly, expression of all GFP-SHARPIN variants was similar and no degradation products were observed (S1A Fig). Taken together, the UBL domain residue V267 is essential for SHARPIN interaction with the α–integrin cytoplasmic domain, whereas the RNF31-binding [6] I272 residue is dispensable for SHARPIN-integrin interaction.

### SHARPIN^V267A^ does not inhibit integrin activity or affect cell migration

**Fig 4:**
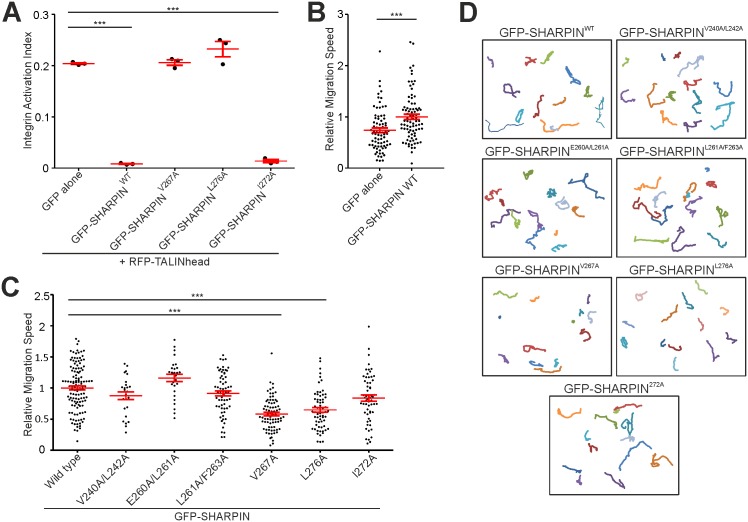
Residues V267 and L276 are essential for SHARPIN-mediated integrin inhibition. (A) FACS analysis of CHO cells overexpressing GFP alone, or WT or mutant GFP-SHARPIN, together with RFP-TALIN head. The Integrin Activation Index was calculated by dividing active cell-surface integrin levels (FN7-10 binding minus FN7-10 binding in the presence of EDTA) by total cell-surface integrin levels (Mb1.2 staining minus secondary antibody alone) (n = 3). (B) Quantification of migration speed of *cpdm* MEFs overexpressing GFP alone or GFP-SHARPIN^WT^ on 50 μg/ml collagen (n = 78 and 83 cells, respectively). (C,D) Quantification of migration speed (n = 27–125 cells) (C), and representative cell tracks (D) of *cpdm* MEFs overexpressing WT or mutant GFP-SHARPIN on 50 μg/ml collagen. All numerical data are mean ± s.e.m. ***: p<0.001, *: p<0.05.

As direct interaction of SHARPIN with integrin results in integrin inactivation [4], we sought to determine the ability of the integrin binding-deficient SHARPIN mutants to inhibit integrin activity using a well-established FACS assay [14,15]. The integrin activation index was measured using fluorescently-labelled fibronectin repeat 7–10 in Chinese Hamster Ovary (CHO) cells overexpressing GFP alone, WT or mutant GFP-SHARPIN, together with RFP-TALIN head to increase integrin activation. Importantly, expression of GFP-SHARPIN variants and RFP-TALIN head was similar in the different samples (S1B Fig). In full agreement with the binding data (Fig 3), expression of GFP-SHARPIN^V267A^ did not result in integrin inactivation (Fig 4A). In contrast, expression of the RNF31-binding mutant GFP-SHARPIN^I272A^ inhibited integrin activity as efficiently as GFP-SHARPIN^WT^ (Fig 4A). Interestingly, despite only slightly reduced ability to bind integrins (Fig 3A–3C), the ability of GFP-SHARPIN^L276A^ to inactivate integrins was completely abolished (Fig 4A), suggesting that, while integrin binding is clearly necessary [4], integrin binding is not sufficient for SHARPIN to inactivate integrins. Alternatively, GFP-SHARPIN^L276A^ might only partially bind integrins and therefore be unable to inactivate integrins.

We have previously shown that SHARPIN regulates cell migration in a substrate concentration-dependent manner, i.e. increased integrin activity induced by SHARPIN silencing decreases cell migration at high substrate concentrations (50 μg/ml collagen) [4]. Therefore, to determine if defects in integrin inhibition translate to changes in cell migration, *cpdm* MEFs expressing GFP alone, WT or mutant GFP-SHARPIN, growing on 50 μg/ml collagen, were imaged using time-lapse microscopy. As expected on this substrate, cells expressing GFP-SHARPIN^WT^ migrated with higher velocity than cells expressing GFP alone (Fig 4B), consistent with GFP-SHARPIN inhibiting integrin activity. In line with the integrin activity assays (Fig 4A), cells expressing GFP-SHARPIN^V267A^ or GFP-SHARPIN^L276A^ migrated significantly slower than cells expressing GFP-SHARPIN^WT^ (Fig 4C and 4D), while all other mutants affected cell migration in a similar fashion as GFP-SHARPIN^WT^. Taken together, these data show that SHARPIN^V267A^ is not only unable to bind integrins, but is also unable to inhibit integrin activity or affect cell migration.

### The RNF31 and integrin binding sites in SHARPIN partially overlap

**Fig 5:**
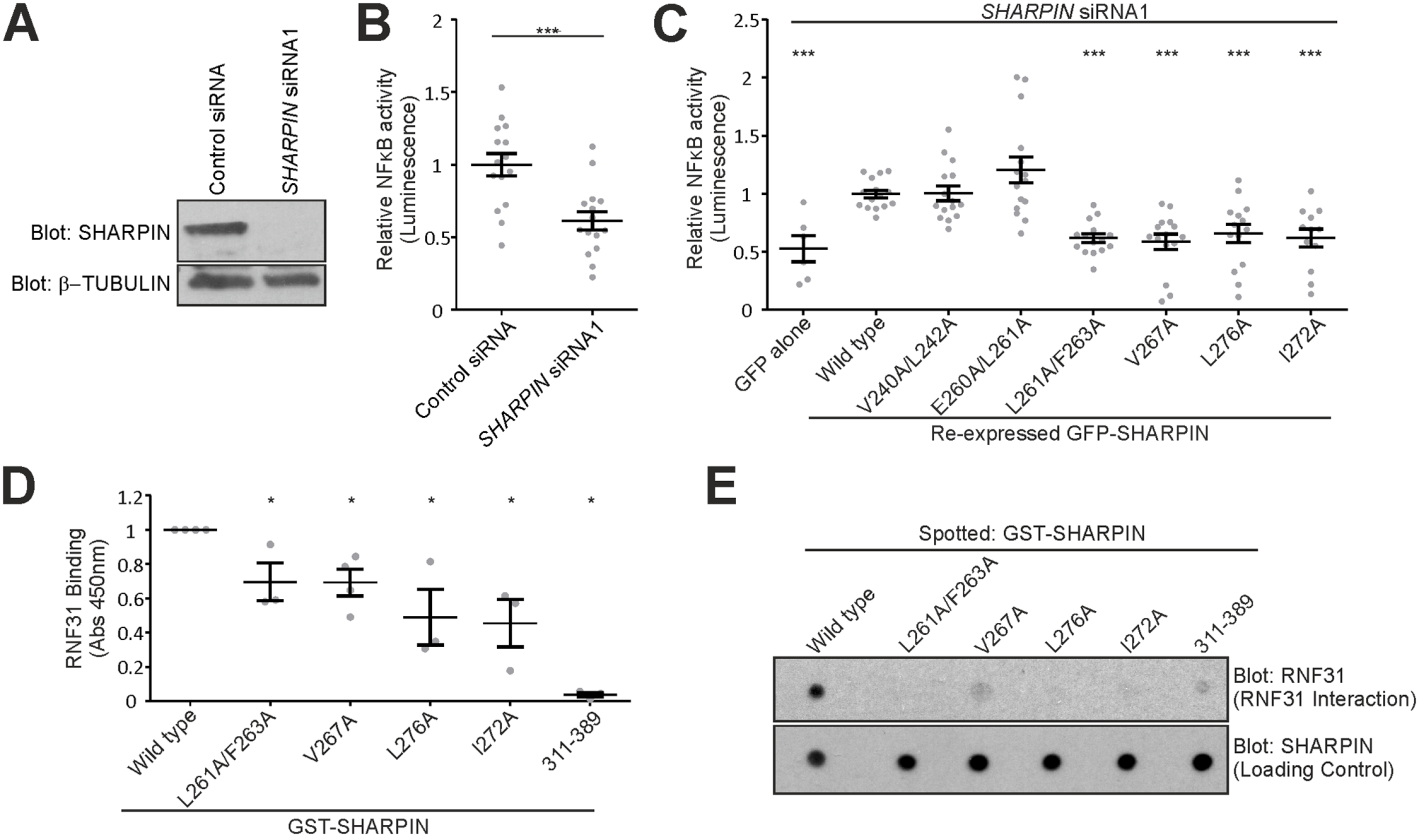
Fine mapping of the RNF31 binding site in SHARPIN. (A) Western blot analysis of SHARPIN and β-tubulin levels in control- or SHARPIN-silenced PC3 cells. (B) TNF-induced NF-κB promoter activity of SHARPIN- or control-silenced PC3 cells was measured using a luciferase reporter assay. (n = 3 with 5 replicates each). (C) TNF-induced NF-κB promoter activity of SHARPIN-silenced PC3 cells, expressing GFP alone, WT or mutant GFP-SHARPIN (n = 6–15 measurements from 2–3 experiments). (D,E) Interaction between RNF31 and WT or mutant GST-SHARPIN was determined using an ELISA-based binding assay (n = 3) (D) or Far-Western analysis (E). All numerical data are mean ± s.e.m. ***: p<0.001, **: p<0.01, *: p<0.05.

**Fig 6:**
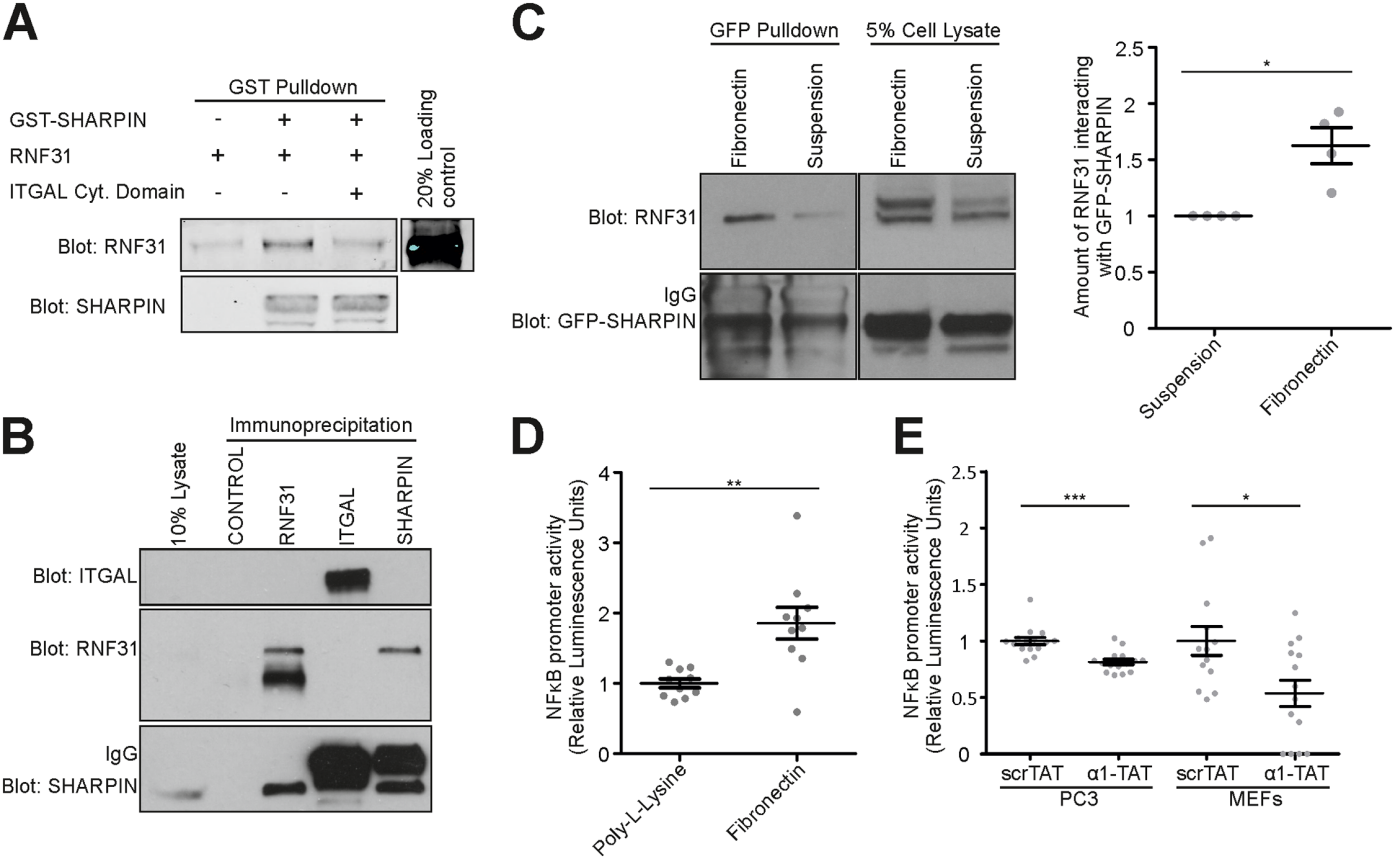
Integrin and RNF31 binding to SHARPIN are mutually exclusive. (A) Pull-down assay showing that the presence of a peptide corresponding to the cytoplasmic domain of ITGAL prevents interaction between GST-SHARPIN and RNF31. (B) Co-immunoprecipitation of endogenous SHARPIN, RNF31 and ITGAL from Jurkat cells (a GFP antibody was used as negative control). (C) Pull-down of GFP-SHARPIN from HEK293 cells demonstrated that cells in suspension show decreased RNF31-SHARPIN interaction compared to adherent cells (n = 4). RNF31 binding was normalized to total RNF31 levels and the amount of pulled-down GFP-SHARPIN. (D) TNF-induced NF-κB promoter activity of PC3 cells, adherent to either 5 μg/ml fibronectin or poly-L-lysin (a substratum for integrin-independent cell adhesion), was measured using a luciferase reporter assay (n = 2 with 5 replicates each). (E) TNF-induced NF-κB promoter activity of PC3 cells or WT MEFs, incubated with a membrane-permeable ITGA1-tail peptide (α1-TAT) or a scrambled peptide (ScrTAT), was measured using a luciferase reporter assay (n = 2 with 6–9 replicates, and n = 3 with 3–5 replicates, respectively). All numerical data are mean ± s.e.m. ***: p<0.001, **: p<0.01, *: p<0.05.

Our data show that residues V267 and L276, but not I272, are involved in SHARPIN-mediated integrin inactivation, indicating that the RNF31 and integrin binding sites are not identical, although both are located within the SHARPIN UBL domain (Fig 1 and [6]). Previous reports have demonstrated a role for SHARPIN in Tumor Necrosis Factor (TNF, also known as TNFα) induced NF-κB activity in MEFs, keratinocytes, B-cells and hepatocytes [6–8,16], as well as in activated B-cell like diffuse large B-cell lymphoma [17], PC3 and DU145 prostate cancer cells [18]. We now confirm that siRNA-mediated SHARPIN silencing in PC3 cells (Fig 5A) reduced TNF-induced NF-κB activity as measured using a dual luciferase assay (Fig 5B).

Next the ability of the SHARPIN mutants to support LUBAC activity was tested (Fig 5C). As expected, re-expression of GFP-SHARPIN^WT^ in SHARPIN-silenced PC3 cells rescued NF-κB activity significantly compared to expression of GFP alone (Fig 5C). GFP-SHARPIN^L261A/F263A^, GFP-SHARPIN^V267A^, GFP-SHARPIN^L276A^ and GFP-SHARPIN^I272A^, on the other hand, were unable to rescue NF-κB activation (Fig 5C), indicating a role for these residues in LUBAC function. ELISA-based binding assays (Fig 5D) and Far-Western analyses (Fig 5E) using recombinant RNF31 and WT or mutant GST-SHARPIN showed that the impaired ability of these mutants to support LUBAC function corresponds to reduced SHARPIN-RNF31 interaction. Therefore, in addition to the previously reported I272 [6], residues F263, V267 and L276 are required for SHARPIN binding to RNF31 and are thus essential for LUBAC function. In summary, our data show that the RNF31 and integrin binding sites partially overlap (around V267), with the RNF31 binding site covering a larger area on SHARPIN. Importantly, SHARPIN mutants F263A and I272A can be used to specifically disrupt LUBAC function without affecting the ability of SHARPIN to regulate integrin activity.

### Integrin and RNF31 binding to SHARPIN are mutually exclusive

**Fig 7:**
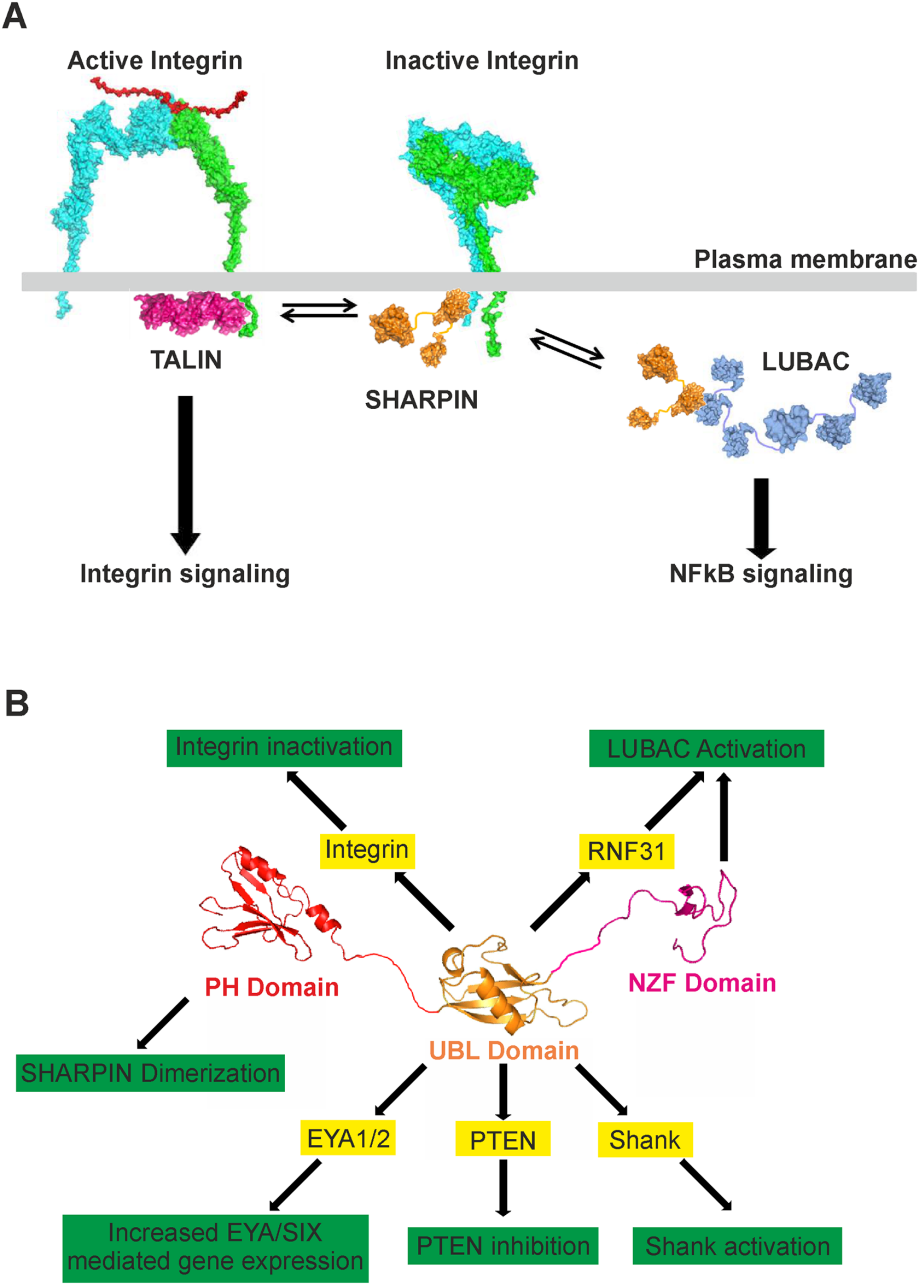
Current model of SHARPIN function. (A) SHARPIN inhibits integrin activation by binding to the α-integrin cytoplasmic domain and preventing binding of the integrin activator TALIN. In addition, SHARPIN is part of LUBAC, which is required for activation of the canonical NF-κB pathway. We now demonstrate that integrin and RNF31 binding are mutually exclusive as they are mediated by partially overlapping binding sites within the SHARPIN UBL domain. (B) An overview of SHARPIN interactions and how these interactions affect diverse signaling pathways. SHARPIN is depicted schematically with its functional domains (an N-terminal PH domain, a central UBL domain and a C-terminal NZF domain). The UBL domain is a multi-faceted protein interaction hub that has been shown to interact with a number of proteins, highlighted in yellow bars. These interactions each have specific functional consequences, highlighted in green bars. In addition, the N-terminal PH domain of SHARPIN mediates SHARPIN homodimerization and the C-terminal NZF domain is required for LUBAC function.

Our data showing that the integrin binding site is nested within the RNF31 binding site suggest that integrin and RNF31 might compete for SHARPIN binding. Indeed, the ability of GST-SHARPIN to bind recombinant RNF31 in vitro was inhibited in the presence of a SHARPIN-binding peptide [5] corresponding to the cytoplasmic domain of ITGAL (Fig 6A). Importantly, integrin-bound endogenous SHARPIN, immunoprecipitated using an ITGAL antibody from Jurkat cells, did not associate with RNF31 (Fig 6B), further demonstrating that integrin-bound SHARPIN is not able to simultaneously bind RNF31 and vice versa. SHARPIN interacts preferentially with the unengaged integrins in cells in suspension and is displaced from activated integrins in adherent cells [4]. Consistent with the mutually exclusive RNF31 and integrin binding, we found increased interaction between GFP-SHARPIN and RNF31 in adherent HEK293 cells compared to cells in suspension (Fig 6C; normalized to total RNF31 levels and the amount of GFP-SHARPIN in pull-downs). Furthermore, TNF-induced NF-κB activity is higher in PC3 cells adherent to fibronectin compared with cells adherent to poly-L-lysine, to which cells adhere in an integrin-independent manner (Fig 6D), suggesting that the increased RNF31-SHARPIN interaction in adherent cells (Fig 6C) correlates with higher LUBAC activity. Lastly, treatment of PC3 cells or MEFs with a membrane permeable TAT-peptide corresponding to the cytoplasmic tail of ITGA1 (α1-TAT), which contains the SHARPIN binding site and is capable of sequestering cytoplasmic SHARPIN in cells [4], diminished TNF-induced NF-κB activation compared to cells treated with a scrambled TAT-peptide (ScrTAT) (Fig 6E). All-in-all, these experiments suggest that SHARPIN binds integrin and RNF31 in a mutually exclusive manner and that SHARPIN might act as a functional link between the interdependent integrin and NF-κB signaling pathways (see model in Fig 7A).

## Discussion

SHARPIN plays a role in integrin inhibition [4,5] and regulation of LUBAC activity [6–8], but if and how these seemingly distinct SHARPIN functions are related has remained unknown. This study now demonstrates that integrin binds to the conserved UBL domain of SHARPIN, which also mediates SHARPIN interaction with RNF31 [6]. Molecular modeling and mutational analysis showed that the integrin binding site is nested within the RNF31 binding site. In line with this, RNF31 and integrin binding to SHARPIN are mutually exclusive *in vitro* and in cells, demonstrating that the roles of SHARPIN in inhibiting integrin activity and supporting linear ubiquitination are molecularly distinct (see model in Fig 7A).

SHARPIN function is not limited to regulation of integrin and NF-κB activity. SHARPIN was originally identified as an interactor of Shank proteins in the post-synaptic density [10]. SHARPIN binding to the N-terminal ARR (ankyrin repeat region) domain of Shank activates Shank through prevention of an intramolecular, inhibitory interaction with the SPN (SHANK/ProSAP N*-*terminal) domain [19]. In addition, SHARPIN directly binds EYA1 and EYA2 (eyes absent homolog 1 and 2), which enhances EYA-SIX complex-mediated activation of target gene expression in several tissues during development [20], suggesting a nuclear function for SHARPIN. Furthermore, direct interaction of SHARPIN with PTEN (phosphatase and tensin homolog) inhibits the phosphatidylinositol 3,4,5-trisphosphate (PIP3) phosphatase activity of PTEN [21]. Thus, SHARPIN is clearly a multifunctional protein (Fig 7B) with most likely scaffolding or adaptor protein-like functions, as SHARPIN has no demonstrated enzymatic activity. Interestingly, all these known SHARPIN regulatory functions are mediated via direct binding of these proteins to the SHARPIN UBL domain [6,10,20,21], indicative of its role as a multi-faceted protein interaction hub. Given the relatively small size of the UBL domain (Fig 2) it is unlikely that all these SHARPIN interactors bind simultaneously. In line with this, we show that SHARPIN binding to integrin and RNF31 is mutually exclusive due to partially overlapping interaction sites. The set of SHARPIN UBL domain mutants described in this study can be utilized as a tool to determine whether additional SHARPIN interactors also occupy the RNF31 and integrin interaction site. Furthermore, mutants that specifically disrupt one SHARPIN function but leave others intact, such as the RNF31-binding SHARPIN^L261A/F263A^ and SHARPIN^I272A^ mutants that are fully functional as integrin inhibitors, will be instrumental in the future to distinguish between distinct functions of SHARPIN. Two decades ago cell adhesion-induced integrin activation was shown to increase NF-κB activity [22,23], although the molecular mechanism has remained obscure. It is attractive to hypothesize that SHARPIN mediates this phenomenon as SHARPIN is released from integrins upon cell adhesion [4]. However, while the data presented here support such a model (Fig 6C–6E), this remains to be investigated further.

The ability of SHARPIN to regulate multiple cellular functions via the central UBL domain suggests that different signaling pathways may compete for SHARPIN and, thus, that SHARPIN functions as a signaling co-ordinator. Which SHARPIN function dominates over the others is likely to be cell-type and context dependent and also regulated by additional cellular components. One such cellular component could be filamin, an integrin inhibitor that forms a ternary complex with both the α- and β-integrin cytoplasmic domains [24]. The SHARPIN and filamin binding domains in the integrin α-tail, while very close, do not overlap [4,24], opening the possibility that filamin and SHARPIN bind integrins simultaneously and possibly cooperatively, resulting in stronger inhibition of integrin activity. On the other hand, as the α-integrin cytoplasmic domains are very small, SHARPIN and filamin could equally well bind in a mutual exclusive fashion, in which case high filamin levels would release SHARPIN from integrins. Which of these possibilities is correct and whether filamin regulates other SHARPIN functions remains to be investigated. In another example, SHARPIN enhances PTEN Lys63-linked polyubiquitination, which in turn increases interaction between PTEN and SHARPIN [25], although it remains unclear what triggers PTEN ubiquitination. At present, whether competition for SHARPIN dictates the balance of these different signaling pathways is unknown as there are no data available on the relevant stoichiometry of SHARPIN and its interacting proteins. However, this implies a possible scenario where acute regulation of SHARPIN levels by some external stimuli could potentially switch the balance between SHARPIN-regulated pathways in favour of the highest affinity interactor.

Deregulation of integrins, PTEN and the NF-κB pathway are all strongly linked to cancer progression [1–3,26–28]. Importantly, SHARPIN is amplified in a wide variety of human cancer types (BioPortal.org; [29]). Furthermore, SHARPIN protein levels are elevated in prostate cancer [18] and SHARPIN promotes cancer cell proliferation and tumor formation in several different cancer types [18,21,30]. In DU145 prostate and HeLa cervical cancer cell lines these oncogenic effects were attributed to SHARPIN’s ability to inhibit PTEN function [21]. However, in PTEN-null PC3 prostate cancer cells SHARPIN also increased tumorigenicity implying that other functions, such as NF-κB signaling, are also involved [18,30]. Thus, while SHARPIN clearly has oncogenic properties, the molecular mechanisms remain poorly described and are most likely dependent on the other malignant signaling alterations present. Some of the SHARPIN mutants described in this study could be utilized to determine how the distinct SHARPIN functions contribute to cancer progression.

## Material and Methods

### Antibodies

The following antibodies were used in this study: RNF31/HOIP (ab46322, Abcam; 1:1000 western blot), GST (91G1, Cell Signaling Technology; 1:1000 western blot), GFP (A11122, Molecular Probes; 1:1000 western blot), RFP (Invitrogen; 1:3000 western blot), SHARPIN (ab69507, Abcam; 1:300 western blot), Anti-hamster α5β1 PB1 antibody (Developmental Studies Hybridoma Bank; 1:100 FACS), β-tubulin (ab6160, Abcam; 1:1000 western blot).

The secondary antibodies used in this study were Alexa 488- or Alexa 564-conjugated IgGs (immunofluorescence; Invitrogen) and HRP-conjugated IgGs (Far-Western analysis, ELISA assay and immunoblotting; GE Healthcare).

### Plasmids

Construction of GST-SHARPIN and siRNA1-insensitive GFP-SHARPIN has been described [4]. The SHARPIN point mutations were introduced in these vectors using standard site-directed mutagenesis. For construction of the GFP- and GST-SHARPIN fragments, the corresponding DNA fragments were amplified from siRNA1-insensitive GFP-Sharpin using specific primers that introduce XhoI and XbaI (GFP-SHARPIN) or EcoRI and BamHI sites (GST-SHARPIN), followed by cloning into pEGFPC1 (Clontech) and pGEX4T-1 vectors, respectively. The sequence integrity was verified for all constructs.

The ITGA5-mCherry and RFP-TALIN head expression constructs were kind gifts from K. Yamada (National Institutes of Health) and D. Calderwood (Yale University), respectively. The GST-RNF31 construct [7] was generously provided by H. Walczak (University College London). The pGL4.32[luc2P/NF-κB-RE/Hygro and pRL-TK *Renilla* luciferase control vectors were purchased from Promega.

### Synthetic Peptides and Recombinant Proteins

Synthetic, biotinylated peptides corresponding to the cytoplasmic domains of human ITGAL and ITGB2 or the conserved membrane proximal sequence of ITGAL (αL-CytD; YKVGFFKRNLKEKMEA) were obtained from Lifetein. The membrane-permeable ITGA1-tail peptide (α1-TAT) and scrambled peptide (ScrTAT) were from Lifetein. Recombinant full-length human ITGAL-ITGB2 was from Origene. Recombinant GST-RNF31 and GST-SHARPIN (WT, fragments and point mutants) were produced in the *E*. *coli* strain Rosetta BL21DE3 and purified according to manufacturer’s instructions (BD Biosciences). The GST-moiety of GST-RNF31 was then cleaved off using PreScission Protease (GE healthcare) according to manufacturer’s instructions.

### Protein Interaction Analysis using Fluorescence Polarization

Fluorescence polarization assays were performed as described previously [4].

### Cells and Transfections

Jurkat, PC3 and HEK293 cells (all from American Type Culture Collection) were grown in DMEM (Jurkat) or RPMI1640 medium (PC3 and HEK293) supplemented with 1% penicillin-streptomycin, 10% fetal bovine serum (FBS), and 1% L-glutamine. CHO cells (American Type Culture Collection) were grown in MEM Alpha Medium + 5% FBS. The generation and maintenance of *cpdm* MEF cells has been described [4]. Plasmid and siRNA transfections were done using Lipofectamine 2000 (Life Technologies) and Hiperfect (Qiagen) respectively.

### Immunoblottings, Immunoprecipitations, Pull-Downs and Far Western Assays

All immunoblottings, immunoprecipitations, pull-downs and far western assays were performed as described previously [5].

### FACS

Chinese Hamster Ovary (CHO) cells expressing RFP-TALIN head, together with GFP, or WT or mutant GFP SHARPIN, were labelled with Alexa 647 fluorescently-labelled fibronectin repeat 7–10 (FN7-10) [31] as described [4]. As a negative control FN7-10 binding in the presence of 10 mM EDTA was used. For normalization purposes, cells were stained using an antibody recognizing total hamster ITGA5-ITGB1, followed by fluorescently conjugated secondary antibodies. Samples were analyzed using FACSCalibur and CellQuest software (BD Biosciences).

### Molecular Modeling

Molecular modelling was performed using Swiss-Model server [12] (PDB access: 1v5oA; sequence identity: 25.71%; E-value: 7.6e^-17^). Protein structure visualization and graphics was done using the PyMOL Molecular Graphics System, Version 1.5.0.4 (Schrödinger, LLC). Color-coding according to residue hydrophobicity in Fig 2 was based on the Kyte-Doolittle hydrophobicity plot as follows: Light pink: Asn, Asp, Gln, Glu, Lys, Arg; Violet: Thr, Ser, Pro, His; Light magenta: Ala, Gly, Cys, Tyr; Hot pink: Ile, Leu, Val, Phe, Met, Trp.

### FRET measurements by FLIM

Fluorescence Lifetime Imaging Microscopy (FLIM) analysis of Fluorescence Resonance Energy Transfer (FRET) and data analysis were performed as previously reported [32].

### Cell Migration Assay

For migration assays *cpdm* MEFs on 50 μg/ml collagen were transfected with GFP alone, or WT or mutant GFP-SHARPIN expression plasmids. The next day a GFP fluorescence image was taken to identify the GFP-positive cells, followed by phase-contrast imaging at 10 min intervals for at least 8 h. Imaging was done using a Zeiss inverted wide-field microscope (x10 objective) equipped with a heated chamber (37°C) and CO_2_ controller (4.8%). Image processing and cell tracking (MTrackJ plugin) were done with NIH ImageJ software.

### NF-κB Reporter Assay

PC3 cells were silenced for SHARPIN using Hs_*SHARPIN*_1 HP siRNA (Qiagen) as described [4] and 48 h later plated on a 96-well plate. The next day these cells were transfected with Renilla Luciferase control vector (pRLTK), NF-κB reporter plasmid (pGL4.32[luc2P/NF-κB-RE/Hygro]) and WT or mutant GFP-SHARPIN expression plasmids. A GFP-only expression vector was used as a negative control. The next day, medium was replaced with medium with or without 50 ng/ml TNF, and after 5 h the luciferase activity was measured using the Dual-Luciferase Reporter Assay System (Promega), according to manufacturer’s instructions, on the ENVISION 2100 multilabel plate reader (Perkin Elmer) (n = 4, 3 wells per condition).

For the TAT-peptide competition experiment, PC3 cells transfected with pRLTK and pGL4.32[luc2P/NF-κB-RE/Hygro] were pre-incubated with 250 mM membrane-permeable SHARPIN binding-tail peptide (α1-TAT) or scrambled peptide (ScrTAT) [4] for 30 min. Subsequently, cells were incubated for 5h with or without 50 ng/ml TNF and 250 mM α1-TAT or ScrTAT, before being processed as described above (n = 2 with 6–9 replicates for PC3 cells and n = 3 with 3–5 replicates for WT MEFs).

For the adhesion vs suspension experiment, PC3 cells transfected with pRLTK and pGL4.32[luc2P/NF-κB-RE/Hygro] were plated in serum free, antibiotic free medium on wells coated with 5 μg/ml fibronectin (Sigma) or 10 μg/ml poly-L-lysine (Sigma) for 16 h. Subsequently, cells were incubated for 5 h with or without 50 ng/ml TNF in serum free, antibiotic free medium, before being processed as described above (n = 2 with 5 replicates each).

### ELISA Assay

Recombinant RNF31 was coated on 96-well plates (Corning, NY, USA) overnight at +4°C. After blocking with blocking buffer (0.1% Tween20, 5% BSA, 10 μM ZnCl_2_ and 1 mM DTT in Tris-buffered saline (TBS)), wells were incubated 3 h at room temperature with 0.1 mM of recombinant WT or mutant GST-SHARPIN in blocking buffer. After washing with blocking buffer, samples were incubated for 1 h with anti-GST antibody in blocking buffer, followed by 1 h incubation with anti-Rabbit horseradish peroxidase (HRP)-conjugated secondary antibody in blocking buffer. HRP activity was detected with the TMB substrate kit and the Multiscan Ascent plate reader (both Thermo Scientific).

### Statistical Analysis

All statistical analyses were performed using GraphPad Prism version 5.03 for Windows (GraphPad Software, San Diego California USA, www.graphpad.com). The Student's t-test was used for normally distributed data (Shapiro-Wilk normality test alpha = 0.05). For all other experiments the Mann-Whitney test was used. A p < 0.05 was considered significant.

## Supporting Information

S1 Fig Expression levels of WT or mutant GFP-SHARPIN. (A) Western blot analysis of WT or mutant GFP-SHARPIN in HeLa cells. (B) Western blot analysis of GFP alone or WT or mutant GFP-SHARPIN in CHO cells. Also the levels of RFP-TALIN head were determined. Non-transfected CHO cells were used as control.
(TIF)
