# Supplementary figures and images for: Mutually Exclusive Roles of SHARPIN in Integrin Inactivation and NF-κB Signaling
Source: PLoS One. 2015 Nov 23;10(11):e0143423. doi: 10.1371/journal.pone.0143423 (PMC4658161; doi:10.1371/journal.pone.0143423)

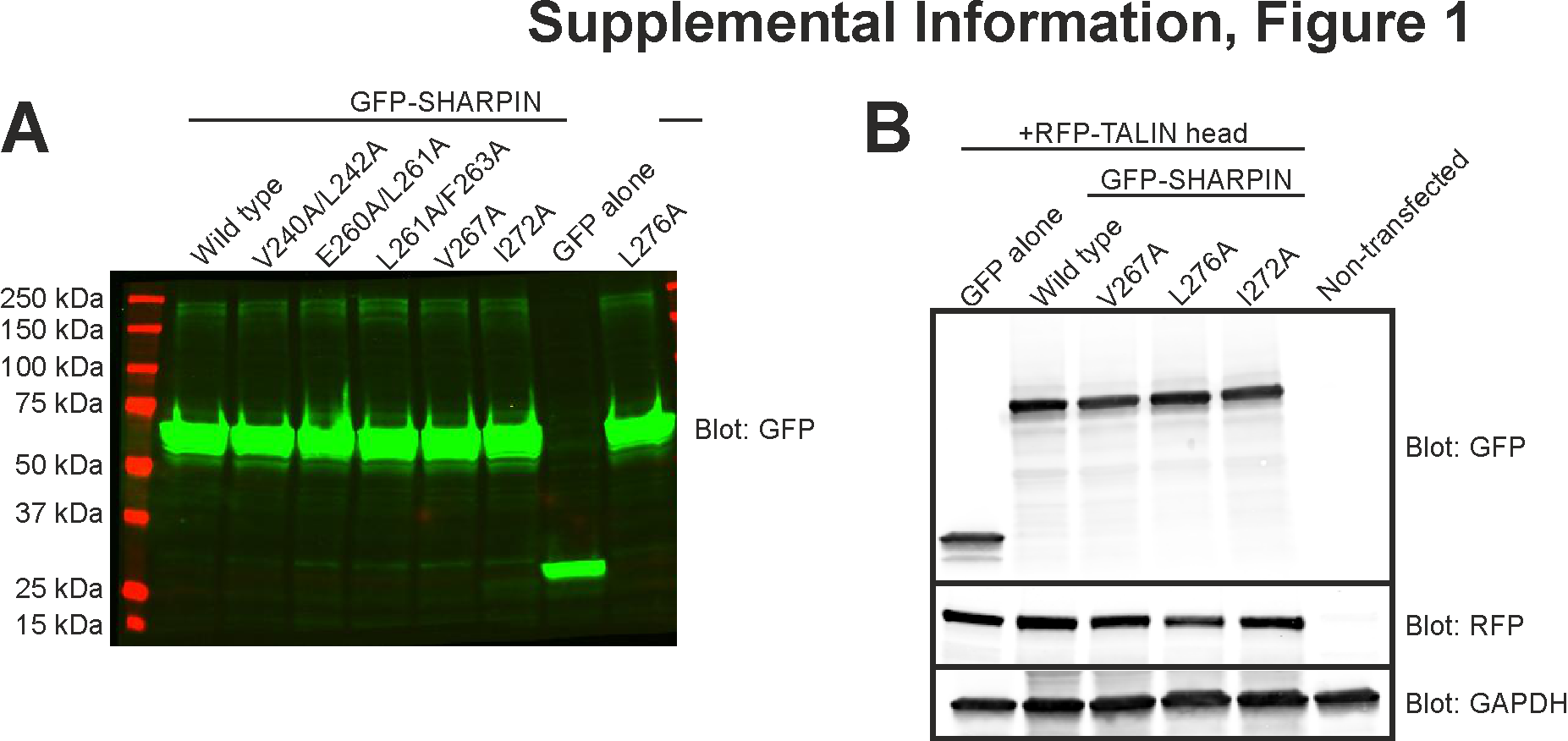

Supplement: S1 Fig — (A) Western blot analysis of WT or mutant GFP-SHARPIN in HeLa cells. (B) Western blot analysis of GFP alone or WT or mutant GFP-SHARPIN in CHO cells. Also the levels of RFP-TALIN head were determined. Non-transfected CHO cells were used as control. (TIF) [file pone.0143423.s001.tif]
